# Clinical and Economic Burdens of ADHD Across Developmental Stages: A Review

**DOI:** 10.3390/jcm15218645

**Published:** 2026-11-06

**Authors:** Farzad Salehpour, Francisco Gonzalez-Lima

**Affiliations:** 1 Texas Consortium in Behavioral Neuroscience, Department of Psychology, The University of Texas at Austin, Austin, TX 78712, USA; 2 Interdisciplinary Neuroscience Program, Department of Psychiatry and Behavioral Sciences, Dell Medical School, The University of Texas at Austin, Austin, TX 78712, USA

**Keywords:** ADHD, economic burden, cost of illness, healthcare utilization, family burden, clinical implications

## Abstract

**Background:** Attention-deficit/hyperactivity disorder (ADHD) is a prevalent neurodevelopmental disorder that can persist across the lifespan and is associated with substantial clinical, economic, and psychosocial burden. This narrative review examines the clinical and economic burdens of ADHD on individuals and families, encompassing direct medical costs, including assessment and treatment expenditures, alongside non-medical costs, educational and occupational disruption, caregiving demands, and adverse mental health outcomes among family members. It synthesizes evidence across developmental stages and geographic regions and considers the clinical implications of these interconnected burdens. **Methods:** A structured search of PubMed, Scopus, Web of Science, and Embase, supplemented by reference-list screening, identified English-language publications from January 2000 through June 2026. Evidence was synthesized narratively across direct medical, non-medical, and family-related burdens, prioritizing large population-based and administrative claims studies, longitudinal investigations, systematic reviews, and meta-analyses. Interpretation considered study design, confounding factors, economic perspectives, and differences in cost estimation. **Results:** ADHD is associated with substantial healthcare, non-medical, and family-related economic costs. Reported costs encompass diagnostic assessment, treatment, comorbidity, and adverse events, alongside educational support, occupational losses, accidents, and criminal justice involvement. Families experience reduced occupational productivity, elevated caregiving demands, greater healthcare utilization, and increased depression, anxiety, and stress. **Conclusions:** Clinically, these findings support assessing functional and family-level burden alongside symptoms, identifying and managing comorbidities, maintaining treatment during transitions to adult services, and monitoring tolerability. Integrating these burden domains into routine assessment and treatment planning may help identify modifiable drivers of impairment and guide more comprehensive care across the lifespan for patients and their families.

## 1. Introduction

The conceptualization of attention-deficit/hyperactivity disorder (ADHD) has evolved over time, with increasing recognition of its neurodevelopmental nature, persistence, and functional consequences across the lifespan [1]. ADHD is associated with substantial economic and psychosocial burdens on individuals, families, and society [2,3]. The economic burden encompasses direct medical costs associated with assessment, treatment, and healthcare utilization, alongside costs related to educational support, occupational disruption, and caregiving demands experienced by affected individuals and their families [4,5,6]. Parenting a child with ADHD can require substantial time and effort and is associated with elevated parenting stress, strained family relationships, and poorer parental mental health [7,8,9]. These family burdens can accumulate throughout childhood and adolescence, while educational, occupational, and healthcare costs remain relevant into adulthood [4,6,10].

From an epidemiological perspective, reported ADHD prevalence estimates from international reviews and U.S. population surveys range from approximately 7.6–11.1% in children [11,12], 5.6–14.2% in adolescents [11,13], and 2.5–6% in adults [14,15]. These estimates indicate that ADHD affects a substantial proportion of the population across developmental stages, underscoring the importance of understanding its clinical and economic burdens. From a clinical perspective, economic-burden evidence also provides a complementary view of functional impairment, comorbidity, and caregiver strain and may help identify priorities for assessment, treatment planning, and support across developmental stages.

In the U.S., a systematic review with national cost extrapolation estimated annual excess societal costs of ADHD ranging from US$38 billion to US$72 billion for children and adolescents and from US$105 billion to US$194 billion for adults (2010 U.S. dollars) [2]. In the Netherlands, annual healthcare costs for children and adolescents with ADHD were estimated at €84–377 million (2012 euros), based on extrapolation of cost estimates from European studies [16]. A subsequent systematic review reported annual excess cost estimates for adult ADHD derived from Danish register data and extrapolated to other countries, amounting to US$325.14 million in Denmark, US$3.77 billion in France, US$1.00 billion in the Netherlands, and US$3.72 billion in the United Kingdom (2019 U.S. dollars) [6]. Another published estimate placed the annual societal cost of childhood/adolescent ADHD in the U.S. at US$124.5 billion [4]. Although differences in populations, cost components, monetary years, and extrapolation methods limit direct comparisons, these findings demonstrate substantial economic costs associated with ADHD for individuals, families, and society.

Existing studies document substantial costs associated with ADHD, but differences in populations, healthcare systems, and cost definitions complicate their interpretation across developmental stages and geographic settings [2,6]. This narrative review aims to synthesize evidence on direct medical, non-medical, and family-related economic burdens associated with ADHD and identify their principal cost drivers and clinical implications. Throughout this review, developmental stages and geographic settings are considered as contexts for interpreting variation in reported costs, rather than as factors whose independent effects are formally evaluated. The available evidence is concentrated in school-aged children, adolescents, and working-age adults. Preschool-aged children, emerging adults considered separately from broader adult samples, middle adulthood beyond employment-related outcomes, and older adults are less well represented. The developmental perspective adopted here describes the review’s scope rather than equally comprehensive evidence across all developmental stages. The included economic evidence is concentrated in high-income Western countries. Its applicability to other settings is limited by differences in healthcare financing, access to ADHD assessment and treatment, educational services, disability support, and family caregiving responsibilities.

## 2. Search Strategy and Methodological Approach

This narrative review aims to synthesize evidence on the clinical and economic burdens of ADHD on individuals and families across the lifespan, encompassing direct medical costs, non-medical costs, productivity losses, caregiving demands, and family health burdens. A narrative approach was chosen to integrate a broad and heterogeneous literature spanning different developmental stages, healthcare systems, economic perspectives, and cost domains, while considering its implications for clinical assessment and management.

A structured literature search was conducted in July 2026 in PubMed, Scopus, Web of Science, and Embase, covering publications from January 2000 through June 2026. Database searches were supplemented by manual screening of reference lists from relevant articles, systematic reviews, and key economic studies. Search terms included combinations of “ADHD”, “attention-deficit/hyperactivity disorder”, “economic burden”, “cost of illness”, “healthcare costs”, “medical expenditures”, “healthcare utilization”, “treatment costs”, “non-medical costs”, “educational costs”, “occupational costs”, “productivity loss”, “absenteeism”, “presenteeism”, “unemployment”, “caregiver burden”, “family burden”, “spillover costs”, “parental mental health”, “accidents”, and “criminality”.

English-language publications were selected if they examined economic costs associated with ADHD, healthcare utilization, productivity losses, caregiving demands, or family health outcomes. No geographic restrictions were applied. Psychosocial and clinical studies were included when relevant to family burden or cost drivers; publications outside this scope were excluded. Titles and abstracts were screened, followed by full-text review. Priority was given to large population-based and administrative claims studies, longitudinal investigations, systematic reviews, and meta-analyses quantifying individual and family costs. Earlier studies within the specified publication period were retained when they provided influential estimates or addressed domains with limited recent evidence.

The review was designed as an interpretive narrative synthesis rather than a systematic or scoping review. Accordingly, no PRISMA flow diagram was prepared, and no quantitative pooling or standardized risk-of-bias assessment was performed. Evidence was organized and synthesized narratively across direct medical costs, non-medical economic costs, and family-related burdens. Interpretation considered study design, sample size, ADHD ascertainment, comparator selection, adjustment for comorbidities and other potential confounders, and the economic perspective adopted. Particular attention was given to differences in age groups, geographic settings, healthcare systems, cost components, price years, currencies, and whether estimates represented total or incremental costs. Estimates were presented as reported in the cited sources, with currency and price-year information indicated where available, rather than converted to a common monetary basis. These differences limit direct comparisons across studies. Where multiple publications or reviews drew on the same underlying studies or populations, their estimates were interpreted as potentially overlapping evidence rather than independent findings. Cost estimates were not summed across publications. Because most included studies were observational, excess costs were interpreted as associations with ADHD rather than costs caused by ADHD itself; psychiatric, developmental, medical, and socioeconomic factors may also contribute to the observed differences.

Study selection was guided by relevance to the review’s scope, with attention to variation and inconsistency in reported estimates. Nevertheless, the narrative approach does not establish exhaustive coverage of the literature, and selection bias cannot be excluded. The synthesis therefore provides an interpretive overview of the available evidence rather than a definitive pooled estimate of costs associated with ADHD.

## 3. Clinical Management and Direct Medical Costs Associated with ADHD

This section reviews direct medical costs associated with ADHD assessment, treatment, and healthcare utilization. The discussion focuses on studies from the United States, the United Kingdom, and Germany, which provide detailed cost estimates across age groups and healthcare services and illustrate findings from different healthcare settings. These countries were selected for the relevance and detail of the available evidence, rather than higher ADHD prevalence, and the discussion is not intended as an exhaustive international comparison.

ADHD-specific assessment and treatment costs should be distinguished from all-cause healthcare expenditures among individuals with ADHD, which may also include care for psychiatric and medical comorbidities. Studies differ in whether they exclude, adjust for, or separately examine these conditions. Consequently, excess all-cause costs should not be interpreted as ADHD treatment costs alone, and differences in comorbidity handling may contribute to variation across estimates.

### 3.1. United States

A study investigated the direct medical costs of children (5–11 years) and adolescents (12–17 years) in the U.S. population with ADHD, using claims data from the IBM MarketScan Research Databases for 2017 and 2018. The excess medical costs associated with ADHD were estimated at US$2801 per child and US$3718 per adolescent compared with children and adolescents without ADHD [17]. For national extrapolation, the study used ADHD prevalence estimates of 10.0% for children aged 5–11 years, drawn from the 2018 National Survey of Children’s Health (NSCH), and 6.5% for adolescents aged 12–17 years, drawn from the National Comorbidity Survey Replication Adolescent Supplement (NCS-A), as reported by Schein et al. [17]. The costs amounted to US$5.0 billion for children (accounting for 26% of the total societal excess costs associated with ADHD) and US$4.0 billion for adolescents (accounting for 29%) [17]. These findings are consistent with previous evidence reporting a healthcare cost range of US$621–US$2720 per child and adolescent (ages 0–21 years), with estimated total medical costs ranging from US$4.12 billion to US$18.04 billion, adjusted to 2010 U.S. dollars [2,18,19].

A study involving 1206 adults classified as having ADHD in a U.S. sample estimated medical expenditures associated with ADHD [20]. The estimated per-capita annual incremental medical costs associated with ADHD, including inpatient and outpatient visits, prescription medication, emergency room visits, office-based visits, and home health visits, were US$2591 (2019 U.S. dollars). The main medical cost drivers were prescription medication costs (US$1347), accounting for 52% of incremental medical costs, followed by office-based visits (US$725), which accounted for 28%. Further analysis by age subgroups revealed that among adults aged ≥31 years, the incremental cost associated with ADHD was US$2623, which was almost 41% higher than that of young adults (18–30 years), whose incremental cost was US$1857. The study classified 1.44% of its analytic sample as having ADHD and estimated total annual incremental medical expenditures of US$8.29 billion nationally. This proportion reflects ADHD ascertainment within the data source and should not be interpreted as a population prevalence estimate. Other estimates summarized by Witrick et al. ranged between US$1.32 billion and US$39.79 billion, adjusted to 2019 U.S. dollars [20]. Differences in ascertainment, populations, and estimation methods limit direct comparisons across these estimates.

A study estimated the total annual expected costs for treating school-age children with ADHD using six commonly prescribed pharmacotherapies. The average annual expected cost per person was highest for Adderall (US$2232), followed by Ritalin (US$2080), methylphenidate immediate-release (US$1845), methylphenidate immediate-release/extended-release (US$1792), Concerta (US$1631), and Metadate CD (US$1487). Researchers applied these estimated average costs to an ADHD prevalence range of 3–8% among U.S. children, based on 2000 U.S. population estimates, and found that the total expected cost of pharmacotherapies ranged from US$4.02 billion to US$10.94 billion for Adderall and US$3.74 billion to US$10.19 billion for Ritalin [21]. These historical modeled estimates reflect the medications, prices, and treatment assumptions used in the 2001 analysis and should not be interpreted as current pharmacotherapy costs. Present-day expenditures depend on formulation, brand or generic availability, insurance coverage, and healthcare financing; patients’ out-of-pocket payments also differ from total treatment costs.

Furthermore, a U.S. claims-based study estimated that, for a hypothetical health plan covering one million adults, annual incremental healthcare costs associated with adverse events during adult ADHD treatment episodes totaled US$125.1 million, with anxiety (US$60.4 million), nausea (US$18.1 million), and fatigue (US$14.5 million) accounting for the largest costs (2022 U.S. dollars) [22]. Clinically, this finding supports attention to tolerability during treatment, although the claims analysis could not establish whether medication caused the recorded events or whether changes in prescribing would reduce the associated costs.

### 3.2. United Kingdom

In a retrospective observational cohort study conducted in the UK, involving 3229 individuals with ADHD and 7429 matched controls, researchers identified individuals newly diagnosed with ADHD between 1998 and 2010 and estimated healthcare costs following the index date. In the first year following the index date, mean total National Health Service (NHS) healthcare costs across all ages were approximately four times higher for individuals with ADHD compared to controls (£1327 vs. £328). Among individuals with ADHD, outpatient attendances accounted for 44% of costs, followed by prescriptions (23%), hospital admissions (16%), and primary care appointments (16%). Specifically, for individuals aged 6 to 17 years, first-year NHS healthcare costs were 4.1 times higher than in controls (£1290 vs. £315), while for individuals aged ≥18 years, first-year costs were 3.35 times higher (£1844 vs. £550) [23].

A relatively small UK study of 143 adolescents with ADHD aged 12–18 years assessed healthcare resource use over a 12-month period at a five-year follow-up [24]. The mean annual NHS cost per adolescent was approximately £1310 (2010 prices), comprising £657.94 for healthcare services and £652.08 for medication. Researchers extrapolated this estimated mean healthcare cost to the UK population using ADHD prevalence estimates of 3.62% in boys and 0.85% in girls and mid-2009 population estimates, yielding estimated annual national healthcare costs of approximately £160.80 million [24].

Using a base-case modeling scenario, Schlander projected annual ADHD medication expenditure for children and adolescents in England to reach approximately £78 million in 2012, comprising £58.3 million for stimulants and £20.0 million for non-stimulants (2006 prices) [25]. This was a forecast based on assumptions about future treatment uptake and prescribing patterns, rather than observed 2012 expenditure.

### 3.3. Germany

A study analyzed claims data from a German Statutory Health Insurance database covering 25,300 individuals with ADHD to estimate medical costs and costs associated with comorbidities by age and the demographic variable labeled “gender” in the source study, reported as female or male [26]. All costs were expressed in 2014 euros. Across all age groups, there were surplus medical costs of €1508 in comparison to the costs of care for individuals without ADHD. Annual excess costs averaged €1549 per female and €1467 per male with ADHD across age groups. The main medical cost drivers were hospital costs, which accounted for 43% of the overall surplus costs, followed by psychiatrists at 16%, stimulant medication at 11%, and psychotherapists and occupational therapy. Specific age-based analysis showed that in the ADHD group, costs were highest in the age group ≥31 years, with a surplus cost of €2182 per ADHD patient per year, followed by ages 0–12 years (€1494), ages 13–17 years (€1447), and ages 18–30 years (€1255). Lower healthcare costs among individuals aged 18–30 years, particularly for stimulant medication and psychiatric care, may be consistent with a gap in service use during the transition to adulthood [26,27]. However, comparisons across age groups do not establish that individuals disengaged from care as they aged or identify the reasons for lower expenditure. Data also showed that mood and anxiety disorders, substance use disorders (SUDs), and obesity were significantly more frequent among individuals with ADHD; individuals with ADHD and these conditions incurred up to approximately €2800 higher annual costs per patient than individuals with the same conditions without ADHD (2014 euros) [26]. These findings highlight the importance of assessing continuity of care during emerging adulthood. Lower use of specialist and medication services should not automatically be interpreted as reduced need, although the economic benefits of coordinated transfer require direct evaluation.

## 4. Non-Medical Economic Costs Associated with ADHD

This section organizes non-medical economic costs by burden domain, drawing on relevant studies from different countries. Geographic coverage varies across domains according to the evidence included in the review; the section therefore does not provide a uniform comparison of the countries discussed above.

### 4.1. Academic and School Costs

Families may incur additional out-of-pocket educational expenses, including private tutoring, learning materials, summer classes, and other supplementary learning services. These family-borne expenses should be distinguished from expenditures on services provided by schools and public educational agencies [28]. At the educational-service level, data from the nationally representative British Child and Adolescent Mental Health Survey indicated that mean annual costs per child with an ICD-10 hyperkinetic disorder were approximately £1762 for frontline educational support and £963 for special education (2007/2008 prices). Frontline educational support included parental meetings with teachers, additional assistance from teachers and learning support assistants, contact with special educational needs officers, and involvement in special educational needs tribunals [29]. Similarly, a U.S. study on societal excess costs estimated the annual educational burden of ADHD at US$11.6 billion for children and US$6.7 billion for adolescents. Special education was identified as the primary driver of educational costs in the U.S. analysis [17]. These costs included additional days per year spent on education, expenses related to tutoring, salaries for special education teachers, service providers, and administrators, as well as expenditures on non-personnel items such as materials, supplies, and technological supports. Educational costs accounted for approximately 60% and 49% of total societal excess costs in children and adolescents, respectively, making education the largest cost component in both age groups [17].

At the household level, additional expenses may arise when children with ADHD experience difficulties in organizing materials and lose belongings and school supplies, necessitating replacement [30]. Families may also incur expenses for missed lessons or extracurricular activities for which they have already paid fees or purchased materials and equipment [4]. These expenses are distinct from school and public educational-service expenditures.

### 4.2. Occupational Costs

In Australia, the total annual social and economic costs of ADHD were estimated at US$15,664 per individual (2018–2019 U.S. dollars), including financial costs and monetized losses in wellbeing. Productivity losses, including absenteeism, presenteeism (reduced productivity while at work), reduced employment, premature mortality, and informal caregiving, accounted for approximately 81% of financial costs [31]. Mortality-related productivity losses were valued using expected future earnings discounted at 2% annually. Separately, the wellbeing component included years of life lost, monetized using a value-of-a-statistical-life-year approach based on willingness to pay to reduce mortality risk [31].

A review of U.S. studies reported annual productivity-related costs ranging from US$209 to US$6699 per employee with ADHD (2010 U.S. dollars), encompassing absenteeism, presenteeism, disability payments, and/or workers’ compensation, with presenteeism identified as a major cost driver for employers [2]. A nationally representative U.S. study of workers aged 18–44 years found that ADHD was associated with approximately 35 lost workday-equivalents annually per worker, encompassing absenteeism and presenteeism, with estimated national productivity losses of US$19.5 billion annually [32]. A separate U.S. cost-of-illness analysis estimated annual excess costs of US$66.8 billion for unemployment and US$28.8 billion for productivity losses among adults with ADHD (2018 U.S. dollars) [33]. That analysis separately estimated US$3.2 billion in mortality-related productivity losses using a human-capital approach based on estimated excess deaths, age-specific employment rates, and earnings, with a 3% annual discount rate [33]. These estimates reflect different methods and cost components and should not be summed.

### 4.3. Accident-Related Costs

Individuals with ADHD have a higher rate of road traffic accidents, potentially resulting in increased family burdens and expenses, including vehicle damage, traffic tickets, and higher automobile insurance costs [4,34]. Separately, a U.S. claims-based study estimated the incidence and costs of accidental injuries broadly, identified through medical claims for injury or poisoning, among children younger than 12 years, adolescents aged 12–18 years, and adults; these estimates were not specific to road-traffic accidents [35]. Among adults, accident-specific direct medical costs, prescription drug costs, and indirect work loss costs (e.g., disability and absenteeism) were significantly higher for individuals with ADHD compared to controls (US$483 vs. US$146). However, no significant differences in accident-specific costs were observed between the ADHD and control groups for children (US$113 vs. US$126) and adolescents (US$178 vs. US$130). Accident-related costs were higher among adults with ADHD than among children or adolescents with ADHD; however, these comparisons do not establish whether differences in accident severity, treatment intensity, or other factors explain the age pattern [35].

### 4.4. Criminality Costs

Criminal justice involvement and associated costs among individuals with ADHD should be interpreted in the context of co-occurring conduct problems, substance use, social disadvantage, and other developmental risk factors [36]. As summarized by Matza et al. [37], a study reported in a 2001 conference presentation by Swensen et al. [38] assessed criminality-related costs in a relatively small historical cohort of participants with and without childhood ADHD who were aged 19–25 years at follow-up. Mean total costs incurred by victims and the criminal justice system were US$12,868 among participants with childhood ADHD versus US$498 among controls, representing an incremental cost of US$12,370 per participant (2004 U.S. dollars) [37]. Barkley [39], citing the same original study [38], reported that this incremental cost accrued over a 13–15-year follow-up. Because the original presentation could not be retrieved, adjustment for relevant confounders could not be independently verified. These cumulative estimates should not be interpreted as costs independently caused by ADHD or as contemporary population-level criminal justice costs.

Beyond their financial implications, educational disruption, work difficulties, accidents, and legal involvement may provide information about functional difficulties not captured by symptom counts alone. These findings suggest potential priorities for assessment and coordination among clinicians, families, schools, employers, and social services [40,41]. However, cost-of-illness evidence does not establish that specific coordination or treatment strategies improve these outcomes or reduce costs.

## 5. Family-Borne Economic Burdens of ADHD

### 5.1. Family-Borne Occupational Burdens

Working parents or caregivers of individuals with ADHD may miss work for various reasons, including attending in-school meetings, pursuing or revising special education services, accompanying children to physician visits, or addressing accidents or legal issues. Parental absenteeism and reduced productivity may accompany reduced working hours, employment changes, and financial strain; these observational findings do not establish that the child’s ADHD itself caused the observed differences [42]. A Dutch study estimated annual productivity losses from absenteeism and reduced efficiency at work at €2243 per employed caregiver of a child with ADHD, compared with €674 among employed caregivers of children without behavioral problems, approximately a 3.3-fold difference (2004 euros) [43].

### 5.2. Family-Borne Health Burdens

Family members of individuals with ADHD experience a substantial mental health burden, particularly parents and caregivers. A meta-analysis found higher depressive symptom levels among mothers of children with ADHD than among mothers of children without ADHD (d = 0.58, 95% CI 0.43–0.74; 18 studies) [44]. These findings establish an association between child ADHD and maternal depressive symptoms but do not establish that caregiving caused those symptoms. Beyond parents, a UK study found that siblings of individuals with ADHD reported lower overall life satisfaction and greater exposure to sibling bullying [45]. Collectively, these findings align with broader evidence indicating reduced quality of life and elevated psychopathology among families of children with ADHD, including higher levels of depression, anxiety, and stress [8,46,47].

These findings require interpretation in the context of familial liability. Parents and siblings may themselves have ADHD symptoms or diagnoses, and shared genetic and environmental vulnerabilities may contribute to psychiatric difficulties within families [9]. Caregiving demands, such as supervision, appointment attendance, and school coordination, should therefore be distinguished from relatives’ mental-health conditions and healthcare utilization [10]. These burdens may coexist, but observed differences between families do not establish how much is attributable to caregiving rather than pre-existing psychopathology or shared familial vulnerability [9,10].

Beyond psychosocial difficulties, family members of individuals with ADHD may experience greater healthcare utilization and associated medical expenditures. A U.S. employer-based claims analysis involving 3404 family members of children with ADHD, none of whom had a recorded ADHD diagnosis themselves, and 3933 family members of matched control children found that the former group had 1.6 times as many medical claims, primarily reflecting greater use of office and outpatient services and mental disorder-specific care. Annual average combined direct and indirect costs were also almost twice as high among family members of children with ADHD (US$2728 vs. US$1440 per family member, 1998 U.S. dollars), encompassing medical and prescription drug expenditures and work-loss costs [42]. Although this analysis examined family members without a recorded ADHD diagnosis, this does not exclude unrecognized ADHD symptoms or establish that differences were independent of other familial psychopathology. A separate U.S. analysis estimated annual excess healthcare costs among family members at US$5.1 billion for family members of children and adolescents with ADHD and US$9.1 billion for family members of adults with ADHD (2000 U.S. dollars) [48].

### 5.3. Spillover Healthcare Costs Among Family Members

Spillover medical costs refer to additional healthcare expenditures for family members of individuals with ADHD compared with family members of individuals without ADHD, regardless of insurance coverage or who pays these costs. Here, “spillover” describes observed excess healthcare expenditures among family members and does not establish that these costs were caused by caregiving. Shared familial liability and relatives’ own health conditions may also contribute to these differences. In U.S. studies, these additional healthcare costs among family members have been reported to range from US$1088 to US$1658 per family member of children and adolescents with ADHD (ages 0–18 years) [42,48] and US$1051 per family member of adults with ADHD (ages 19–44 years) [48], adjusted to 2010 U.S. dollars. Using these per-family-member costs, U.S. population estimates, ADHD prevalence assumptions of 7.2% for children and adolescents and 4.4% for adults, and household-size multipliers, Doshi et al. estimated annual excess healthcare costs among family members at US$16.97–25.86 billion for families of children and adolescents with ADHD and US$14.62 billion for families of adults with ADHD (2010 U.S. dollars) [2].

## 6. Synthesis of ADHD-Related Economic Burden Estimates and Cost Drivers

Prevalence estimates used in economic analyses may vary according to population characteristics, age definitions, diagnostic criteria, and ascertainment methods. Their interpretation should also consider ongoing debate about diagnostic expansion, including changes in diagnostic thresholds, differences between DSM and ICD criteria, and cross-national variation in diagnostic practices [49,50]. Accordingly, the population-level economic estimates discussed in this review should be interpreted alongside the prevalence assumptions and diagnostic definitions used in the underlying studies.

In the U.S., a systematic review with national cost extrapolation estimated annual excess societal costs of ADHD ranging from US$38 billion to US$72 billion for children and adolescents and from US$105 billion to US$194 billion for adults (2010 U.S. dollars) [2]. Using cost estimates derived from Danish register data, Daley et al. extrapolated an annual economic burden of US$126.2 billion for adult ADHD in the United States, assuming an adult ADHD prevalence of 2.5% and applying Danish per-person costs to the U.S. population [51]. This estimate falls within the range reported by Doshi et al. [2], although differences in methods limit direct comparison. Variation across studies reflects differences in populations, prevalence assumptions, cost components, healthcare systems, and approaches to accounting for family characteristics and psychiatric comorbidity [52].

More recent U.S. cost-of-illness analyses provide age-specific estimates of the societal burden of ADHD. For children, total annual excess societal costs were estimated at US$19.4 billion, while for adolescents, costs reached US$13.8 billion. In both age groups, education costs accounted for the largest share of total excess costs (US$11.6 billion [~60%] in children; US$6.7 billion [~49%] in adolescents), followed by direct healthcare costs (US$5.0 billion [~26%] in children; US$4.0 billion [29%] in adolescents) and caregiving costs (US$2.7 billion [~14%] in children; US$1.6 billion [11.5%] in adolescents) [17]. Moreover, in another U.S. analysis of adult ADHD, excess societal costs were estimated at US$122.8 billion annually, with the majority attributable to indirect costs totaling US$105.4 billion (~86%), including unemployment, productivity loss, caregiving, and premature mortality. Excess direct healthcare costs accounted for US$14.3 billion (~11.5%), largely driven by pharmacy and medical service expenditures, while excess direct non-healthcare costs totaled US$3.1 billion (2.5%), including road traffic accidents, disability, SUDs, and research and training [53]. These findings indicate that the greatest economic burden in the U.S. for children and adolescents is associated with education and healthcare, while for adults, the largest cost burden is attributable to income and productivity losses, underscoring the need for targeted interventions and support systems to address the unique financial challenges faced by different age groups.

The substantial family burden associated with having a child with ADHD encompasses financial, occupational, social, and mental health consequences, including direct medical costs (e.g., expenses related to ADHD assessment and treatment), direct non-medical costs (e.g., childcare, academic support, and legal services), lost work time or employment, stress, anxiety, depression, and relationship strain (e.g., marital tension) [10,40]. In a U.S. longitudinal sample recruited in western Pennsylvania, families with a child with ADHD experience a 6.5-fold greater socio-occupational burden and costs related to caregiver strain over the course of development from childhood through adolescence, compared to control families (US$13,659 per child vs. US$2082 per child) [4]. ADHD symptoms and associated cognitive and behavioral difficulties may help contextualize these family burdens, but the reported cost differences do not establish their independent contributions [54]. For instance, these families reported approximately 1.8 times the behavior-related costs (e.g., academic challenges, lost belongings, missed activities, damaged property, accidents/injuries, and legal involvement) of families without a child with ADHD (US$1378 per child vs. US$766 per child) [4]. Overall, the total annual economic burden of ADHD on U.S. families has been estimated at US$5.8 billion (adjusted to 2017 U.S. dollars), underscoring the importance of addressing the direct, indirect, and psychosocial burdens associated with caregiving for individuals with ADHD [4].

Table 1 summarizes the key economic findings discussed in this review, together with study characteristics, economic perspectives, currencies and price years, and main limitations. Table 2 maps the developmental coverage of major burden domains, distinguishing stage-specific findings from estimates derived from broader age groups and highlighting areas with limited evidence in the included literature.

**Table 1:** Summary of key findings and economic burden estimates associated with ADHD.

Study and Year	Country	Study Design and Sample	Population and Developmental Stage	Burden Domain and Perspective	Cost Period, Currency, and Price Year	Key Economic Findings	Main Limitations
National Cost-of-Illness Modeling Studies							
Birnbaum et al. (2005) [48]	U.S.A	Cost-of-illness study using 1996–1998 employer claims; 1219 individuals with ADHD and 3692 family members; National extrapolation; controls matched on age, sex, employment status, and location; no comorbidity adjustment reported	Children/adolescents aged 7–18 and adults aged 19–44 years; family members aged <65 years; ADHD identified by ≥1 medical claim (ICD-9-CM 314.0x); controls without ADHD claims	Patient and family healthcare expenditures and medically related work-loss costs; National economic perspective incorporating healthcare and employer costs	Annual costs for 2000; underlying observations from 1998; standardized to national healthcare/wage averages and inflated to 2000 U.S. dollars	Modeled annual national excess costs totaled US$31.6 billion, comprising US$1.6 billion for ADHD treatment, US$12.1 billion for other patient healthcare, US$14.2 billion for family healthcare, and US$3.7 billion for patient and family work loss. Family healthcare costs comprised US$5.1 billion among family members of children/adolescents with ADHD and US$9.1 billion among family members of adults with ADHD.	Single employer’s insured population; claims-based diagnoses and limited control for comorbidities; assumed equal excess non-ADHD healthcare and work-loss costs for treated and untreated populations; excluded presenteeism, education, criminal justice, and broader earnings losses
Schein et al. (2022) [17]	U.S.A	Cost-of-illness study using MarketScan claims and published/government data; national extrapolation to 2.86 million children and 1.66 million adolescents; claims controls matched on demographics, insurance, and index year; no comorbidity adjustment reported for the claims comparison	Children aged 5–11 and adolescents aged 12–17 years; claims-defined ADHD required ≥2 diagnoses on distinct dates (ICD-9-CM 314.0x/ICD-10-CM F90.x); claims comparator without ADHD	Direct healthcare, education, and other non-healthcare costs, caregiving, and other indirect costs; Societal perspective	Annual costs; 2018 U.S. dollars; claims data from 2017 to 2018; earlier monetary estimates adjusted using the Consumer Price Index	Modeled national annual excess societal costs were US$19.4 billion for children (US$6799 per child) and US$13.8 billion for adolescents (US$8349 per adolescent). Education accounted for US$11.6 billion and US$6.7 billion, healthcare for US$5.0 billion and US$4.0 billion, and caregiving for US$2.7 billion and US$1.6 billion, respectively.	Estimates depend on prevalence assumptions and heterogeneous published inputs; claims omit undiagnosed ADHD; uncertainty in national extrapolation and generalizability
Schein et al. (2022) [33]	U.S.A	Cost-of-illness study (using 2017–2018 MarketScan claims and published/government data); National extrapolation to 8.7 million adults with ADHD; claims controls matched on demographic and insurance characteristics; no comorbidity adjustment reported for the claims comparison	Adults aged 18–64 years; claims-defined ADHD required ≥2 diagnoses on distinct dates; comparators without ADHD or general-population estimates where unavailable	Direct healthcare, direct non-healthcare, and indirect costs; Societal perspective	Annual costs for 2018; 2018 U.S. dollars; earlier monetary estimates adjusted using the Consumer Price Index	Modeled national estimates: US$122.8 billion in annual excess societal costs, equivalent to US$14,092 per adult with ADHD; comprising US$14.3 billion in healthcare costs, US$3.1 billion in non-healthcare costs, and US$105.4 billion in indirect costs. Unemployment and workplace productivity losses were the largest contributors.	Estimates depend on prevalence assumptions and heterogeneous data sources; potential diagnostic misclassification and limited generalizability to adults aged ≥65 years
Sciberras et al. (2022) [31]	Australia	Prevalence-based cost-of-illness modeling using published and administrative data; Estimated 814,500 individuals with ADHD; Comparators and comorbidity adjustments varied by cost domain	Children, adolescents, and adults across the lifespan; prevalence derived from Global Burden of Disease estimates and Australian ADHD persistence data; no single diagnostic ascertainment method	Healthcare, productivity, education, criminal justice, taxation-related economic losses, and monetized loss of wellbeing; Societal perspective	Annual costs for 2018–2019; earlier costs updated where applicable; converted from Australian dollars using 2019 purchasing power parity (A$1.43 per US$1)	Modeled national annual costs were US$12.76 billion (US$15,664 per person), comprising US$7.45 billion in financial costs and US$5.31 billion in monetized wellbeing losses. Productivity losses accounted for approximately 81% of financial costs; healthcare costs totaled US$321.1 million.	Estimates sensitive to prevalence and modeling assumptions; incomplete healthcare cost coverage; difficulty separating ADHD-related costs from comorbidity-related costs; limited international generalizability
Observational Studies of Healthcare, Service Use, and Family or Productivity Costs							
Guevara et al. (2001) [18]	U.S.A	Healthcare utilization and cost study (retrospective matched cohort using health-plan records); 2992 children with ADHD and 11,968 age- and sex-matched controls; Models accounted for mental health and other medical comorbidities	Children and adolescents aged 3–17 years; ADHD identified by ICD-9 code 314 or stimulant dispensing; controls without ADHD	Direct healthcare costs; Healthcare-system perspective	Annual costs for 1997; 1997 U.S. dollars; no inflation adjustment reported	Unadjusted health-plan costs: Mean annual healthcare costs were US$1465 per child with ADHD versus US$690 without ADHD. Adjusted annual incremental costs were US$375 for ADHD alone and US$812 for ADHD with coexisting mental health disorders.	Single regional health plan; potential diagnostic misclassification; exclusion of out-of-pocket, educational, and family indirect costs
Swensen et al. (2003) [42]	U.S.A	Retrospective employer claims analysis; 1086 children with ADHD and 1086 age- and sex-matched controls, plus 3404 and 3933 respective family members without ADHD; No comorbidity adjustment	Children and adolescents aged ≤18 years, mean age 12.4 years, and their family members; ADHD identified by ≥1 medical/disability claim (ICD-9 314.0x); controls without ADHD claims	Patient and family healthcare expenditures and family-member work-loss costs; Employer perspective	Annual costs in 1998 U.S. dollars; ADHD identified using claims from 1996 to 1998; no inflation adjustment reported	Unadjusted annual direct medical costs were US$1574 per child with ADHD versus US$541 among controls. Annual combined direct and indirect costs were US$2728 per family member without ADHD of a child with ADHD, versus US$1440 among control family members. Family-member costs comprised direct medical expenditures of US$2060 versus US$1026 and work-loss costs of US$668 versus US$414.	Single employer’s insured population; claims-based diagnoses; observational associations; excluded presenteeism and incompletely captured caregiving-related work absence
Swensen et al. (2004) [35]	U.S.A	Retrospective employer insurance claims analysis; 1308 individuals with ADHD and 1308 matched controls; Cost comparisons unadjusted; separate accident-risk models included mental health comorbidity	Children < 12, adolescents 12–18, and adults 19–64 years; ADHD identified by ≥1 medical/disability claim (ICD-9 314.0); controls without ADHD claims	Accident-related healthcare and employee work-loss costs; Employer perspective	Annual costs in 1998; 1998 U.S. dollars; no inflation adjustment reported; claims from 1996 to 1998 used to identify ADHD	Unadjusted mean annual accident-related costs were US$209 per individual with ADHD versus US$131 among controls. Among adults, costs were US$483 versus US$146; differences were not statistically significant among children or adolescents. Among individuals with an accident claim, accident-related costs did not differ significantly between groups.	Single employer’s insured population; claims-based diagnoses; captures only injuries receiving medical attention; work-loss costs limited to employees; observational comparisons
Kessler et al. (2005) [32]	U.S.A	Nationally representative cross-sectional survey; 2399 employed/self-employed respondents; national extrapolation; Adjusted for age, sex, race/ethnicity, education, and occupation, but not comorbidities	Working adults aged 18–44 years; DSM-IV-based ADHD status imputed from a clinical interview subsample; comparator workers without ADHD	Occupational productivity losses from absenteeism and presenteeism; human-capital valuation using salary plus fringe benefits	Annual estimates extrapolated from preceding-month work performance; survey conducted in 2001–2003; U.S. dollars; price year and inflation adjustment not reported	Regression-estimated annual losses associated with ADHD were 35.0 workday-equivalents per worker, valued at US$5661. National extrapolation yielded approximately 120 million lost workday-equivalents and US$19.5 billion in annual productivity losses.	Cross-sectional associations; self-reported work performance; imputed ADHD diagnoses; potential confounding by comorbidities; restricted to workers aged 18–44
Secnik et al. (2005) [55]	U.S.A	Retrospective matched analysis of MarketScan claims and employment records; 2252 adults with ADHD and 2252 controls; work-loss analysis included 354 matched pairs; Cost models adjusted for demographics and psychiatric/medical comorbidities	Continuously insured adults aged 18–64 years; ADHD identified by ICD-9 codes 314.00/314.01; controls without ADHD matched on age, sex, and geographic area; work-loss subsample continuously employed	Direct medical and indirect work-loss costs; Insurer/patient payments and employer costs	Annual costs during the diagnosis year, using 1999–2001 data; medical costs standardized to 2001 U.S. dollars using the medical component of the Consumer Price Index	Adjusted annual medical costs were US$5651 per adult with ADHD versus US$2771 without ADHD; reported incremental cost approximately US$2881. In the employee subsample, annual work-loss costs were US$7044 versus US$5214, respectively.	Limited generalizability beyond continuously insured/employed populations; claims-based diagnostic identification; some absenteeism inferred from medical visits; presenteeism excluded
Hakkaart-van Roijen et al. (2007) [43]	Netherlands	Questionnaire-based cost-of-illness study; 65 children with ADHD, 33 with behavioral problems, and 47 without behavioral problems, plus caregivers; Unmatched comparisons; psychiatric comorbidity examined in subgroups, without multivariable adjustment	Children with ADHD, mean age 10.5 years, and caregivers, predominantly mothers; ADHD diagnosed by a multidisciplinary team using DSM-IV criteria	Child and caregiver healthcare costs and caregiver productivity losses; Societal perspective	Annualized costs in 2004 euros; data collected during 2002–2004; healthcare use valued using 2004 unit prices; no separate inflation adjustment reported	Unadjusted annual child medical costs were €2040 for ADHD versus €288 for behavioral problems and €177 without behavioral problems; excluding one prolonged hospitalization reduced the ADHD estimate to €1173. Caregiver medical costs were €728, €202, and €154, respectively. Annual productivity losses among employed caregivers were €2243 for the ADHD group versus €674 for the group without behavioral problems.	Small, unmatched groups differing in age and sex; caregiver-reported resource use and annualization assumptions; costs sensitive to one hospitalization; behavioral-problem comparison group included children with ADHD
Jones et al. (2009) [19]	U.S.A	Service utilization and cost study (longitudinal cohort); 650 participants, including 84 with ADHD; analyses adjusted for study site, sex, and race; Conduct disorder examined separately	Adolescents aged 12–17 years; ADHD identified by parent diagnostic interview at ages 12 or 15; comparisons by ADHD and conduct-disorder status	Healthcare, medication, school, and juvenile justice costs; Public-sector perspective	Cumulative over six years; data collected during 1997–2004; CPI-adjusted 2000 U.S. dollars	Service-use-based cost estimates: Six-year service costs exceeded US$40,000 per adolescent with ADHD, approximately US$25,000 more than for those without ADHD. Costs exceeded US$80,000 for adolescents with ADHD and conduct disorder, more than twice those for ADHD alone.	Reliance on caregiver reports and imputation of missing data; limited measurement of service intensity; incomplete coverage of broader societal costs
Holden et al. (2013) [23]	United Kingdom; linked hospital data restricted to England	Retrospective CPRD and linked hospital-record study; 3229 individuals with ADHD and 7429 controls; Matched on birth year, sex, and general practice; no comorbidity adjustment reported	Children, adolescents, and adults; mean age 10.4 years; ≥2 recorded ADHD diagnoses or ≥1 diagnosis plus ADHD medication; controls without ADHD diagnoses or medication	All-cause direct healthcare costs, including primary care, prescriptions, investigations, outpatient visits, and hospital admissions; NHS perspective	ADHD presentation during 1998–2010; annual costs during the first five years after presentation; British pounds; prescription costs inflation-adjusted to 2011 prices, with service costs drawn from 2009 to 2011 sources	First-year mean healthcare costs were £1327 per individual with ADHD versus £328 per control. Corresponding costs were £1290 versus £315 among those aged 6–17 years and £1844 versus £550 among adults. Outpatient visits accounted for 44% of first-year costs in the ADHD group.	Potential under-recording of ADHD and specialist care; secondary-care prescriptions excluded; small adult subgroup and possible confounding by comorbidities
Telford et al. (2013) [24]	United Kingdom; participants recruited from clinics in England and Wales	Parent-reported service use at five-year follow-up of the Cardiff Longitudinal ADHD Study; 143 adolescents; no non-ADHD comparison group; Descriptive cost estimates without comorbidity adjustment	Adolescents aged 12–18 years; mean age 14.1 years; childhood ADHD confirmed using a diagnostic interview and DSM-IV or ICD-10 criteria	Mental health-related healthcare, medication, social care, and educational support costs; Public-sector payer perspective	Previous 12 months assessed at follow-up; British pounds, 2010 prices; earlier unit costs inflated to 2010 where applicable; survey calendar years not reported	Service-use-based mean annual cost was £5493 per adolescent (95% CI £4416–£6679), including approximately £4155 for education, £1310 for NHS services and medication, and £28 for social care. Education accounted for approximately 76% of costs. National extrapolation estimated annual costs of approximately £670 million.	Small clinical sample, parent-reported service use, and no comparison group; excluded family costs, emergency services, and criminal justice costs; limited generalizability to untreated or undiagnosed ADHD
Snell et al. (2013) [29]	Great Britain	Longitudinal national survey; 2461 children followed up, including 445 with psychiatric disorders; Costs weighted for survey design and nonresponse; no comorbidity adjustment reported	Children aged 5–15 years at baseline, followed for three years; ICD-10 hyperkinetic disorders identified using the Development and Well-being Assessment; reported estimates are within-group costs, not excess costs versus controls	Psychiatric disorder-related healthcare, frontline, and special education, and social care costs; Public-sector perspective	Three-year costs and annual equivalents; 1999–2002 survey data; British pounds, 2007/2008 prices; earlier unit costs inflation-adjusted	For hyperkinetic disorders, service-use-based mean annual public-sector costs were £3108 per child, including £1762 for frontline education and £963 for special education. Mean cumulative costs over three years were £9324 (95% CI £4610–£14,039).	Parent-reported service use, selective attrition, missing-data imputation, and wide confidence intervals; medication costs not explicitly included and criminal justice costs excluded
Daley et al. (2019) [51]	Denmark; additional modeled extrapolations to other countries	Cross-sectional national-register cost-of-illness study; 460 adults with ADHD and 460 same-sex sibling controls; Psychiatric comorbidity addressed through exclusion rather than regression adjustment	Adults aged 18–65 years in 2010, diagnosed at ages 18–50 using ICD-10 codes F90.x/F98.8; no recorded childhood ADHD or psychiatric comorbidity; mean age 31.7 years; siblings without psychiatric diagnoses	Income, taxation, public benefits, healthcare, medication, education, and criminal justice; Individual and public-sector perspectives	Annual costs for 2010, expressed in euros; conversion at DKK 7.45 per euro; no inflation adjustment reported	Annual excess costs totaled €20,134 per adult with ADHD, comprising €7718 in individual costs and €12,416 in public-sector costs. Major components included reduced disposable income, increased public transfers, and reduced tax revenue; excess criminal justice costs were €1012 per person. Extrapolating Danish cost estimates to the U.S. population using 2.5% adult ADHD prevalence yielded approximately US$126.2 billion annually.	Selected sample excluding psychiatric comorbidity and childhood-diagnosed ADHD; cross-sectional design; adult treatment effects not controlled; international extrapolations assume Danish per-person costs apply elsewhere
Zhao et al. (2019) [4]	U.S.A	Cost-of-illness study (retrospective family survey within a longitudinal cohort); 56 ADHD and 30 demographically matched comparison families; separate models adjusted for IQ or oppositional/conduct symptoms	Childhood through adolescence; assessed at ages 14–17; DSM-III-R ADHD diagnosed at ages 4–6; controls without ADHD	Family expenditures, caregiver work losses, and behavior-related costs; family perspective	Cumulative through adolescence; CPI-adjusted 2017 U.S. dollars; survey calendar years not reported	Unadjusted survey estimates: US$15,036 per child with ADHD versus US$2848 per child without ADHD; incremental cumulative family cost of US$12,188, excluding ADHD treatment costs.	Small, single-region sample; retrospective recall; substantial cost variability
Libutzki et al. (2019) [26]	Germany	Retrospective statutory health insurance claims analysis, 2009–2014; 25,300 individuals with ADHD and 25,300 age- and gender-matched controls; Comorbidity subgroup comparisons without multivariable adjustment	Children, adolescents, and adults; age groups 0–12, 13–17, 18–30, and ≥31 years; ADHD identified by ICD-10 codes F90.0/F90.1/F90.8; controls without ADHD	Healthcare and medication costs, therapeutic services and aids, and sickness payments; Statutory health insurance payer perspective	Annual costs in 2014; 2014 euros; no inflation adjustment reported	Mean annual costs were €2236 per individual with ADHD versus €728 without ADHD, an excess of €1508. Excess costs were highest among adults aged ≥31 years (€2182). Individuals with ADHD and SUDs incurred €2715 higher annual costs than individuals with SUDs without ADHD, averaged across age groups.	Claims-based diagnoses may underidentify ADHD and overrepresent severe cases; limited confounding adjustment; exclusion of high-cost patients and out-of-pocket expenses
Witrick et al. (2023) [20]	U.S.A	Healthcare utilization and cost study (pooled cross-sectional analysis of the Medical Expenditure Panel Survey, 2015–2019); 83,776 adults, including 1206 with ADHD; Adjusted for demographics, insurance, Charlson comorbidity index, asthma, anxiety, and mood disorders	Adults aged ≥18 years; subgroup analyses for ages 18–30 and ≥31 years; reported ADHD diagnosis coded ICD-9 314 or ICD-10 F90; comparator without ADHD	Direct healthcare expenditures; Payments by public/private insurers and patients	Annual expenditures estimated from 2015 to 2019 data; 2019 U.S. dollars; inflation-adjusted using Personal Health Care Price Indices	Regression-estimated annual excess medical expenditures were US$2591 per adult with ADHD compared with adults without ADHD, extrapolated to US$8.29 billion nationally. Prescription medication accounted for US$1347 and office-based visits for US$725. Age-specific excess expenditures were US$1857 at ages 18–30 and US$2623 at ages ≥31.	Self-reported conditions and potential ADHD underascertainment; exclusion of institutionalized populations; cross-sectional design; no non-healthcare or indirect costs
Schein et al. (2024) [22]	U.S.A	Retrospective IQVIA claims analysis, 2015–2021; 461,464 patients; Entropy balancing included demographics, treatment characteristics, and Charlson comorbidity index; AE-related comorbidities were not balanced	Commercially insured adults aged ≥18 years receiving ADHD medication; mean age 34.2 years; ≥2 ADHD diagnoses (ICD-10-CM F90.x); treatment episodes with versus without each AE	Medical and pharmacy costs associated with 10 selected adverse events (AEs); Payer perspective	Per patient per month during treatment episodes; 2022 U.S. dollars; inflation-adjusted using the medical component of the Consumer Price Index	Adjusted excess healthcare costs ranged from US$120 to US$1160 per patient per month for episodes with a given AE versus episodes without that AE. Excess costs were US$380 for anxiety, US$709 for nausea, and US$248 for fatigue. Medical costs specifically linked to AE-coded claims ranged from US$57 to US$723 per patient per month.	Claims likely captured more severe AEs; medication causation could not be established; residual confounding and restriction to commercially insured patients limit interpretation and generalizability
Schein et al. (2024) [56]	U.S.A	Retrospective IQVIA claims analysis, 2015–2021; 494,900 adults; entropy balancing for demographics, insurance, ADHD type, and treatment history; Anxiety/depression-related comorbidities not balanced	Commercially insured adults aged ≥18 years receiving ADHD medication; mean age 34.1 years; ≥2 ADHD diagnoses (ICD-10-CM F90.x); comparator ADHD without anxiety or depression	All-cause medical and pharmacy expenditures associated with comorbid anxiety and/or depression; Payer perspective	Twelve months following initiation of a selected ADHD treatment; 2022 U.S. dollars; inflation-adjusted using the medical component of the Consumer Price Index	Weighted annual healthcare costs were US$11,315 among adults with ADHD and anxiety and/or depression versus US$5335 among those without either diagnosis. Reported annual incremental costs were US$3897 for anxiety alone, US$5315 for depression alone, and US$10,274 for both conditions, relative to ADHD without either diagnosis.	Restricted to commercially insured, pharmacologically treated adults; claims-based diagnoses; residual confounding, including differences in other comorbidities; no causal inference
Treatment-Cost Modeling and Expenditure Projections							
Marchetti et al. (2001) [21]	U.S.A	Treatment-cost modeling study (decision analysis using published trials, expert input, and a survey of eight elementary schools); No directly observed patient cohort; Comparison of six pharmacotherapies, without a non-ADHD control group	School-age children with ADHD; response estimates derived from trials requiring confirmed ADHD and excluding comorbidities	Direct treatment and management costs; Payer perspective, supplemented by school medication-administration costs	Annual modeled costs; U.S. dollars, using 2001 drug prices and medical fee schedules; no inflation adjustment reported	Expected annual costs per child were US$1487 for Metadate CD, US$1631 for Concerta, US$1792 for methylphenidate IR/ER, US$1845 for methylphenidate IR, US$2080 for Ritalin, and US$2232 for Adderall. Differences primarily reflected drug acquisition and school dosing costs.	Estimates depended on modeled treatment pathways and response assumptions; comorbidity-related costs and parental productivity losses were excluded; historical prices and treatment patterns limit current applicability
Schlander (2007) [25]	England and Germany	Treatment-cost modeling using demographic, epidemiological, prescribing, and market data; five scenarios per country, calibrated against 1998–2005 data; No patient cohort or non-ADHD comparator	Children and adolescents aged 6–18 years; base-case ADHD prevalence of 6%, assuming DSM-IV criteria; no patient-level comorbidity adjustment	Direct medical costs limited to ADHD medications; NHS perspective in England and statutory health insurance perspective in Germany	Annual expenditure projections through 2012; GBP for England and EUR for Germany; 2006 medication prices and assumed prices for future products; no price increases assumed during the projection period	Base-case annual medication expenditures projected for 2012 were approximately £78 million in England and €311 million in Germany. Extreme scenarios ranged from £49–117 million and €170–456 million, respectively. England’s base-case projection included £58.3 million for stimulants and £20.0 million for non-stimulants.	Projections depend on assumptions about diagnosis, treatment uptake, treatment intensity, and future medication availability and pricing; excludes other healthcare and societal costs and does not assess cost-effectiveness
Reviews and Evidence Syntheses							
Matza et al. (2005) [37]	Predominantly U.S.A; includes some non-U.S. treatment-economic evaluations	Narrative literature review; MEDLINE search, reference screening, and author contact identified 22 sources; no quantitative pooling; comparators and comorbidity adjustments varied across studies	Children, adolescents, adults with ADHD, and family members; diagnostic ascertainment, ages, and sample sizes varied across studies	Medical care, family costs, criminality, accidents, work loss, and treatment cost-effectiveness; perspectives varied across sources	Costs standardized to 2004 U.S. dollars; U.S. costs inflated using medical CPI; non-U.S. costs adjusted using country-specific inflators before currency conversion; annual and longer-term estimates reported separately	Annual medical costs for children with ADHD exceeded those for matched controls by US$503–1343. Adult annual medical costs ranged from US$4929–5651, compared with US$1473–2771 among matched controls. One conference study reported mean criminality-related costs of US$12,868 versus US$498 per participant, a difference of US$12,370; these were not presented as annual costs.	Heterogeneous sources, predominantly U.S. evidence, limited adult and non-medical cost research, inclusion of unpublished conference findings, and no reported formal quality assessment
Doshi et al. (2012) [2]	U.S.A	Systematic review and national cost extrapolation; 19 original studies published during January 1990–June 2011; comparators and comorbidity adjustments varied; adjusted estimates prioritized where sufficient studies were available	Children, adolescents, adults with ADHD, and family members; ADHD ascertainment, age definitions, and sample sizes varied across studies	Healthcare, productivity and income losses, education, criminal justice, and family spillover costs; Societal perspective	Annual incremental costs standardized to 2010 U.S. dollars using CPI, with medical CPI for healthcare costs; extrapolated using national population and prevalence estimates	Extrapolated annual national excess costs ranged from US$143–266 billion, comprising US$38–72 billion for children/adolescents and US$105–194 billion for adults. Major cost categories were healthcare and education for children/adolescents and productivity and income losses for adults. Family spillover costs totaled US$33–43 billion and were included in the overall estimates.	Heterogeneous methods, populations, and cost definitions; sparse evidence for several domains; no formal adjustment for study quality; national estimates sensitive to prevalence and extrapolation assumptions
Le et al. (2014) [16]	Netherlands, using evidence from Belgium, Germany, the Netherlands, Sweden, and the United Kingdom	Systematic review with national extrapolation; seven studies from January 1990–April 2013; estimates applied to 105,552 children/adolescents; mixed total and incremental costs; comparators varied, and confounder adjustment was limited	Children/adolescents aged 7–17 years in the national model, plus adult family members; assumed ADHD prevalence 4.8%; source studies mainly used DSM/ICD diagnoses; one used a behavioral rating scale	Healthcare, education, social services, and family productivity losses; societal perspective	Annual costs in 2012 euros; converted using purchasing power parity and inflated using Dutch Harmonized Consumer Price Indices; national extrapolation using 2011 Dutch population data	Extrapolated annual national costs were estimated at €1.04–1.53 billion, equivalent to €9860–14,483 per child/adolescent with ADHD, including family costs. Education accounted for €648 million (42–62%); patient healthcare costs were €84–377 million. Family healthcare costs were €161 million, and family productivity losses were €143–339 million annually.	Few studies, heterogeneous methods, and a mixture of total and incremental costs; extrapolation across countries and use of German prevalence data; limited adjustment for confounders and incomplete coverage of cost domains
Chhibber et al. (2021) [6]	International; all included studies conducted in high-income countries, predominantly in North America and Europe	Systematic review with narrative synthesis; 44 original studies; searches through December 2020; no meta-analysis; comparators and confounder adjustments varied across studies	Children, adolescents, adults with ADHD, and family members; populations, age definitions, sample sizes, and ADHD ascertainment varied	Healthcare, non-medical costs, productivity losses, education, criminal justice, and family costs; perspectives varied across studies	Annual estimates standardized to 2019 U.S. dollars; country-specific CPI inflation adjustments followed by exchange-rate conversion; publication year used when cost year was unreported	Reported total annual costs ranged from US$831–20,538 per person, while incremental costs ranged from US$244–18,751. National estimates ranged from US$356 million–20.27 billion for total costs and US$12.18 million–141.33 billion for incremental costs. These ranges reflect different populations and cost components rather than directly comparable estimates or a pooled global total.	Substantial methodological heterogeneity; variable coverage of cost domains; limited adult evidence; no studies from low- or middle-income countries; publication year assumed when price year was unreported

Estimates are presented in the currencies and price years indicated and are not directly comparable across studies because populations, perspectives, cost components, comparators, and observation periods differ. Review-level estimates may incorporate primary studies listed elsewhere in the table and should not be added to those estimates. Ranges may represent variation across studies or modeled scenarios rather than confidence intervals. Abbreviations: AE, adverse event; ADHD, attention-deficit/hyperactivity disorder; CI, confidence interval; CPI, Consumer Price Index; CPRD, Clinical Practice Research Datalink; DKK, Danish krone; DSM, Diagnostic and Statistical Manual of Mental Disorders; EUR, euro; GBP, pound sterling; ICD, International Classification of Diseases; IQ, intelligence quotient; IR/ER, immediate-release/extended-release; NHS, National Health Service; SUD, substance use disorder.

**Table 2:** Developmental coverage and evidence gaps across economic burden domains in the included literature.

Developmental Stage	Healthcare and Treatment	Education	Employment and Productivity	Accidents and Criminal Justice	Family and Caregiver Burden
Preschool childhood	Included in some broader pediatric samples; preschool-specific estimates limited [18,26]	Preschool-specific estimates limited	Not generally applicable to children’s own employment; caregiver productivity considered separately	Included within broader childhood accident estimates; preschool-specific findings limited [35]	Early childhood included in retrospective family histories, but costs not isolated for the preschool period [4]
School-aged childhood	Separate child estimates and broader pediatric analyses of healthcare use, treatment, and excess costs [17,18,21,23,26]	Educational support and special education costs reported in child or mixed child–adolescent samples [17,28,29]	Children’s own employment costs generally not applicable	Childhood accident costs reported; criminal justice costs more often examined in older samples [35]	Caregiver productivity, family expenditures, and family healthcare costs reported [4,42,48]
Adolescence	Adolescent healthcare and treatment costs reported separately in several studies [17,19,24,26]	Adolescent educational and school-service costs reported [17,19,24]	Adolescents’ own employment costs less well represented; caregiver productivity included in some broader estimates [17]	Adolescent accident and juvenile justice costs reported [19,35]	Family expenditures, caregiving, and healthcare costs reported, although some estimates combine childhood and adolescence [4,17,42,48]
Emerging adulthood	Young-adult estimates available for ages 18–30, which overlap with but do not precisely isolate emerging adulthood [20,26]	Transition to further or higher education less well represented	Usually included within broader working-age adult estimates [32,33]	Criminality costs reported at ages 19–25, but accumulated over earlier follow-up; accident estimates generally use broader adult groups [35,37]	Stage-specific estimates limited; some family costs included in broader adult analyses [33,48]
Adulthood	Estimates reported for broad adult samples and categories such as ≥31 years; developmental stages within adulthood rarely distinguished [20,26,55,56]	Adult educational costs included in some analyses, without separate estimates for developmental stages within adulthood [51]	Employment, income, and productivity costs reported in broader working-age samples [32,33,51,55]	Adult accident and criminal justice costs reported, with limited differentiation by developmental stage [35,51]	Family healthcare and caregiving costs included in broader adult estimates; stage-specific evidence limited [33,48]
Older adulthood	Some studies include older adults within broad adult categories; separate older-adult estimates limited [20,26]	Limited evidence in the included literature	Limited evidence beyond conventional working-age populations	Separate older-adult estimates limited	Older-adult-specific caregiving and family spillover estimates limited

Developmental-stage labels are used as conceptual categories. Age boundaries vary across the included studies; actual age ranges are specified where relevant, and findings from broader samples should not be interpreted as stage-specific estimates. “Limited evidence” refers to the literature included in this narrative review and does not imply that no relevant studies exist. Cumulative costs reported at follow-up may include expenditures incurred during earlier developmental stages.

## 7. Clinical Implications for ADHD Assessment, Diagnosis, and Treatment

The economic evidence identifies areas of substantial burden but does not by itself establish the effectiveness or cost savings of specific clinical strategies. The implications discussed below represent potential priorities for assessment and care planning; their effects on clinical outcomes and costs require prospective evaluation.

The reviewed evidence on ADHD-related burden highlights the importance of assessing not only symptom severity but also functional impairment, service use, treatment access, school or workplace disruption, accident risk, caregiver time, and the mental health of family members. This broader approach may help identify potentially modifiable burdens for a particular patient and inform treatment goals that extend beyond symptom reduction to functioning, safety, participation, and quality of life [57]. The observed patterns of service use highlight continuity of care as a potential priority during developmental transitions. Costs associated with adverse events draw attention to tolerability and treatment persistence, whereas the transition gap between child and adult services highlights the risk of clinical disengagement during emerging adulthood [22,27].

Clinical evaluation and management of mood and anxiety disorders and SUDs are particularly relevant in ADHD because these conditions occur more frequently among individuals with ADHD than among those without ADHD. Meta-analyses found that adults with ADHD had higher odds of major depressive disorder (OR 4.51, 95% CI 2.44–8.34), anxiety disorders (OR 4.95, 95% CI 3.29–7.46), and substance use disorders (OR 4.60, 95% CI 2.72–7.80) compared with adults without ADHD, although estimates varied significantly across studies [58]. In addition to their clinical burden, these comorbidities are associated with substantial healthcare and non-healthcare costs. A U.S. retrospective claims study of commercially insured adults receiving pharmacological treatment for ADHD found that comorbid anxiety alone, depression alone, and both conditions were associated with additional annual all-cause healthcare costs of US$3897, US$5315, and US$10,274 per patient, respectively (2022 U.S. dollars), compared with adults with ADHD without either diagnosis [56]. A German statutory health insurance claims study found that individuals with ADHD and comorbid SUDs incurred €2715 higher annual healthcare costs per patient in 2014, averaged across age groups, compared with individuals with SUDs without ADHD [26]. Among U.S. adults with ADHD, annual excess direct non-healthcare costs associated with comorbid SUDs were estimated at US$487 million (2018 U.S. dollars), encompassing criminal justice costs, property damage, crime-related productivity losses, and prevention and research expenditures [33]. Integrated approaches that connect pharmacological treatment and psychosocial management with educational and occupational accommodations may help address these clinical and functional needs, although their effects on downstream costs require direct evaluation.

The impact of ADHD at the family level should likewise be treated as part of the clinical burden of ADHD rather than as a less important consequence. Caregiver strain, lost productivity, and elevated mental-health service use among relatives indicate a need to assess caregiver well-being, provide psychoeducation, and involve families in treatment planning when appropriate [10,42]. Family-focused approaches may improve the feasibility and continuity of care, but whether they reduce family spillover costs remains to be established.

These clinical implications should, however, be interpreted cautiously. Most available studies are observational cost-of-illness analyses; therefore, the reviewed associations do not establish that any single management approach (e.g., pharmacotherapy, psychosocial treatment, behavioral interventions, coordinated care, or family-focused support) will produce specific cost savings.

## 8. Limitations of the Evidence Base

The reviewed evidence is heterogeneous in study design, populations, ADHD ascertainment, comparators, economic perspectives, and included cost components. Estimates span different currencies and price years, and historical expenditures and treatment projections may not reflect contemporary prescribing patterns, medication availability, or service delivery. Although some studies adjusted for inflation, this review retained the monetary basis reported by each source rather than standardizing estimates to a common currency and price year. Direct numerical comparisons should therefore be interpreted cautiously.

Many studies relied on administrative claims, which identify recorded diagnoses and reimbursed services rather than all individuals with ADHD or all costs incurred. Coding errors, undiagnosed ADHD, insurance coverage, and selection into care may influence these estimates. Diagnostic criteria and adjustment for psychiatric, developmental, medical, and socioeconomic factors also varied. Consequently, observed excess costs cannot generally be attributed independently to ADHD. Familial aggregation and shared vulnerability further complicate interpretation of relatives’ mental-health outcomes and healthcare expenditures as consequences of caregiving.

Patient, caregiver, household, and societal costs were not consistently distinguished across studies, and these categories may overlap. Healthcare expenditures incurred for patients or relatives do not necessarily represent out-of-pocket payments by families. Some reviews and modeled estimates also draw on overlapping underlying studies. These limitations restrict comparisons across economic perspectives and mean that overlapping estimates should not be summed.

The selected evidence is concentrated in high-income Western countries, limiting its generalizability to settings with different healthcare financing, access to ADHD care, educational services, disability support, and family caregiving arrangements. Developmental coverage is also uneven, with limited stage-specific evidence for preschool-aged children, emerging adults, and older adults, and limited coverage of middle adulthood beyond employment-related outcomes. Longitudinal and intervention-based economic evidence remains limited; comparisons across age groups do not establish individual developmental trajectories, and cost-of-illness studies do not demonstrate that particular clinical strategies produce cost savings.

Finally, this is a narrative review rather than a systematic review. Literature selection was guided by relevance, no standardized risk-of-bias assessment was performed, and exhaustive coverage cannot be assured. Selection and publication biases may therefore influence the synthesis.

## 9. Conclusions and Future Directions

ADHD is associated with a substantial and enduring clinical and economic burden among individuals and families and across healthcare systems, educational institutions, employers, and society. The reviewed evidence highlights the importance of considering functional and family-related consequences alongside healthcare expenditures when characterizing this burden across the lifespan.

Future research should prioritize stronger prospective and intervention-based economic evidence to determine whether interventions targeting these major burden domains can reduce avoidable downstream costs while improving functioning and quality of life for individuals with ADHD and their families. Greater attention is needed to how age, sex, comorbidities, treatment status, socioeconomic context, and healthcare systems influence cost estimates. Further research should address developmental gaps, including costs in preschool-aged children and older adults, transition-specific burdens in emerging adulthood, and healthcare, family, and social costs in middle adulthood. Research should also evaluate whether early diagnosis, sustained evidence-based treatment, and coordinated educational and occupational support reduce long-term expenditures. Cross-country comparisons and prospective economic evaluations could further clarify where preventive investment produces the greatest societal benefit overall.

## Data Availability

No new data were created or analyzed in this study. Data sharing is not applicable to this article.
